# Therapeutic effect of knee extension exercise with single-joint hybrid assistive limb following total knee arthroplasty: a prospective, randomized controlled trial

**DOI:** 10.1038/s41598-024-53891-7

**Published:** 2024-02-16

**Authors:** Takaya Maeda, Eiji Sasaki, Takayuki Kasai, Shigesato Igarashi, Yuji Wakai, Tomoyuki Sasaki, Eiichi Tsuda, Yasuyuki Ishibashi

**Affiliations:** 1 Department of Rehabilitation, Hirosaki Memorial Hospital, 59-1, Sakaizekinishida, Hirosaki, Aomori Japan; 2 Department of Orthopedic Surgery, Hirosaki University Graduate School of Medicine, Hirosaki, Japan; 3 Unaffiliated, Hirosaki, Japan; 4 Department of Orthopedic Surgery, Hirosaki Memorial Hospital, Hirosaki, Japan; 5 Department of Rehabilitation Medicine, Hirosaki University Graduate School of Medicine, Hirosaki, Japan

**Keywords:** Neuroscience, Medical research

## Abstract

The single-joint hybrid assistive limb (HAL-SJ), an exoskeletal robotic suit, offers functional improvement. In this prospective randomized controlled trial, we investigated the therapeutic effects of knee extension exercises using the HAL-SJ after total knee arthroplasty (TKA). Seventy-six patients with knee osteoarthritis were randomly assigned to HAL-SJ or conventional physical therapy (CPT) groups. The HAL-SJ group underwent exercise using the HAL-SJ for 10 days postoperatively, in addition to CPT; the CPT group underwent only CPT. Pain intensity and active and passive knee extension angles were evaluated preoperatively and on postoperative days 1–10 and weeks 2 and 4. Performance tests and Knee Injury and Osteoarthritis Outcome Scores (KOOS) were evaluated preoperatively and at postoperative weeks 2 and 4. Statistical analysis showed that the HAL-SJ group significantly improved active and passive knee extension angles compared with the CPT group. The HAL-SJ group showed immediate improvement in active knee extension angle through day 5. There were no significant differences in results between the performance tests and KOOS. Knee extension exercises with the HAL-SJ improved knee pain and the angle of extension in the acute phase after TKA.

## Introduction

Total knee arthroplasty (TKA) is one of the most effective orthopedic surgeries for patients with end-stage knee osteoarthritis (OA)^1–3^. Surgical invasion of the knee extension mechanism leads to a remarkable reduction in quadricep strength, especially in the acute phase after surgery^3^. This dysfunction, expressed as knee extension lag (EL), is primarily caused by failure of voluntary muscle activation^3–5^. Although clinical guidelines from the American Physical Therapy Association recommend performing high-intensity strength exercises in the acute phase after TKA to improve muscle function^6^, this is not easy to practice because patients experience moderate to severe knee pain during this period^1^. Therefore, painless muscle activation therapy is required.

Recently, a single-joint hybrid assistive limb (HAL-SJ; Cyberdyne Inc., Tsukuba, Japan) has become available for clinical practice. The HAL-SJ perceives bioelectrical signals from the voluntary muscle activation of the wearer and assists knee motion based on that signal^7^. A previous small case series reported that knee extension training with the HAL-SJ after TKA could improve EL and knee motion pain^8^. Furthermore, to demonstrate the effectiveness of HAL-SJ, a prospective randomized controlled trial evaluating various aspects is necessary.

This study aimed to investigate the efficacy of HAL-SJ knee extension exercises in the acute phase after TKA. This study hypothesized that knee extension training with HAL-SJ would (1) improve active and passive knee extension angles immediately after exercise, (2) improve knee function (i.e., greater knee extension strength and gait function), and (3) show greater improvements in activities of daily living (ADL) and quality of life (QOL) than conventional physical therapy (CPT) without HAL-SJ.

## Methods

**Figure 1:**
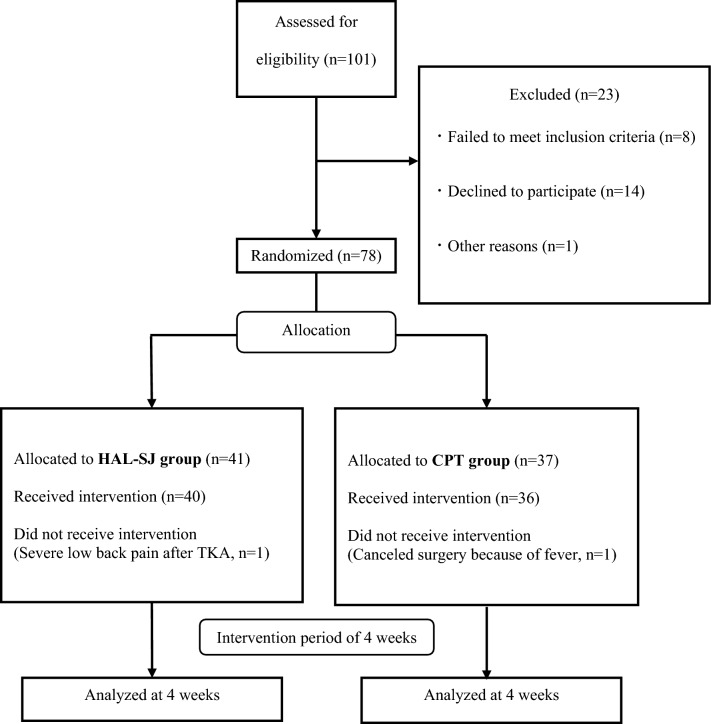
Patient enrollment flow diagram.

This was a prospective, single-center, parallel-group, randomized controlled trial. A total of 101 patients with end-stage knee OA scheduled for primary TKA were enrolled in this prospective study between February 2018 and February 2019. The exclusion criteria were as follows: a history of serious peripheral circulatory disturbances, heart or respiratory problems, central or peripheral neurological disorders, and dementia. Patients with spinal disease complications or lower-extremity symptoms were also excluded. Finally, 76 patients were included in the analysis (Fig. 1). All the patients provided written informed consent to participate in the study. This study was conducted in accordance with the Declaration of Helsinki and was approved by the Hirosaki Memorial Hospital Research Ethics Committee (H29-14). This study was registered to UMIN-CTR on 01/09/2023 (Trial Registration Number: UMIN000052086).

### Randomization and blinding

Participants who met the eligibility criteria were randomly assigned to (1) knee extension exercise using HAL-SJ combined with CPT (HAL-SJ group) or (2) CPT including knee extension exercise without HAL-SJ (CPT: CPT group). Independent of the study, the therapist created a random number table and assigned participants who met the eligibility criteria to the order in which they were admitted. The therapist was informed only of the patient’s ID and was blinded to the baseline evaluation. All participants and their physical therapists were informed of the assigned groups on the day after TKA.

### Intervention

#### Conventional physical therapy

All participants in the HAL-SJ and CPT groups underwent CPT. Physical therapy after TKA started on postoperative day 1. The rehabilitation program was as follows: active and passive knee ROM exercises, quadriceps muscle strength exercises, gait exercises performed on postoperative day 3, and stair-climbing exercises performed on postoperative day 8. All rehabilitation programs were managed by a physical therapist with careful monitoring of the worsening of the pain. Physical therapy was continued until discharge from the hospital at a frequency of five times a week. Participants in the CPT group started active knee extension exercises on postoperative day 4, in addition to the above-mentioned physical therapy. Participants began the exercise in a sitting position, extending the knee joint as much as possible and then flexing it back to the initial knee joint position. During the active knee extension exercises, the physical therapist did not assist with knee joint movement but only provided verbal instructions. These exercises counted as a one-time exercise and were performed 50 times a day. These active knee extension exercises were performed for five sessions per week, and the total number of sessions was 10 (i.e., 10 sessions in 2 weeks).

#### HAL-SJ group

**Figure 2:**
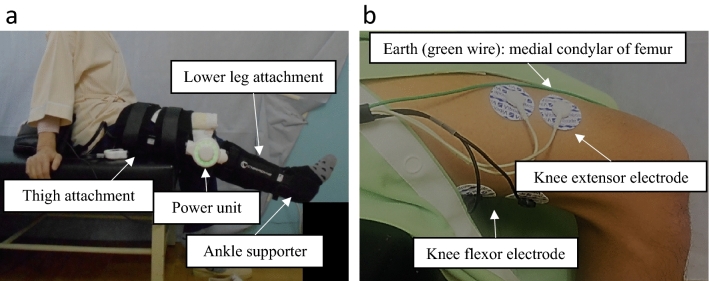
HAL-SJ group set-up. (**a**) Knee extension exercise using the single-joint hybrid assistive limb (HAL-SJ). (**b**) Electrodes were used to detect bioelectrical signals from the knee extensor and flexor.

The patients in the HAL-SJ group performed knee extension exercises in combination with CPT. The HAL-SJ was composed of a power unit, thigh and lower leg attachments, a control device, and a lithium-ion battery (Fig. 2a). The HAL-SJ can perceive bioelectrical signals from the knee extensor or flexor muscles and assist in knee motion according to these signals^7,9^. The electrodes used to perceive bioelectrical signals were set on the skin of the front and rear thighs (Fig. 2b). The HAL-SJ provided visual feedback through the control device via the LED ring positioned on the side of the power unit using various colors according to the bioelectrical signal. Similar to the CPT group, participants began the exercise in a seated position. In the knee extension exercise, the HAL-SJ assisted the knee joint movement according to voluntary muscle activity, and a physical therapist did not assist the joint movement. With the assistance of the HAL-SJ, participants extended the knee joint as far as possible and then flexed the knee joint to return it to the initial knee joint position. The physical therapist immediately informed the participants whether muscle activation during the knee extension exercise was correct. In this study, incorrect muscle activation was defined as knee flexor activation during knee extension and knee extensor activation during knee flexion. The physical therapist adjusted the amount of assistance from the HAL-SJ to achieve full knee extension in each patient. The active knee extension exercises using the HAL-SJ were performed 50 times per day, five sessions per week, as in the CPT group; therefore, the total number of sessions was 10. The number of trials per day was determined based on the report of Goto et al.^8^. The frequency and total number of exercise sessions in exploratory studies were more frequent than those in previous studies. Physical therapy and interventions in the CPT and HAL-SJ groups are described in Supplemental Table S1.

### Surgical procedure

Cruciate-substitute (CS) type TKAs using the Triathlon Knee CS System (Stryker Japan K.K., Tokyo) were performed with the measured resection technique without a navigation system. These surgeries were performed by three senior joint surgeons (ES, YW, and TS). A medial parapatellar approach was used to expose the knees. A femoral osteotomy was performed using an intramedullary guide. A tibial osteotomy was performed using an extramedullary guide with a varus/valgus angle of 0° and a slightly posterior slope. The gap was measured using a tensor. The soft tissue balance was adjusted for straight alignment with minimized medial soft tissue release in a step-by-step manner, such that the medial and lateral gap differences were less than 3 mm at both full extension and 90° flexion. Finally, the components were implanted using a cementless technique.

### Outcome measures

The main outcomes measured in the present study were pain intensity during knee motion and active and passive knee extension angles. Isometric knee extension strength (IKES), gait, standing and sitting abilities, ADL, and QOL were assessed as ancillary outcome measures.

#### Pain intensity

Pain intensity during active knee motion was evaluated using a visual analogue scale (VAS). The VAS is a 100-mm-long horizontal line anchored by word descriptors at each end. The left side of the scale (0 mm) reads "no pain," and the right side of the scale (100 mm) reads "worst pain imaginable.” Each participant drew a vertical mark on the line at the point that best represented their perceived pain.

#### Active and passive knee extension angles

The participants were seated on a bed, and two sagittal plane photographs of each active and passive knee extension were obtained. The knee extension angle was measured using image analysis software (ImageJ; National Institutes of Health, Bethesda, MD, United States)^10^. The knee extension angle analysis was performed by two physical therapists who were blinded to the group assignments. The average value was used for statistical analysis.

#### IKES

Participants were seated in a wheelchair with their hip and knee joints flexed and stabilized at 90°. A handheld dynamometer (Microfet2; HOGGAN Scientific, Salt Lake City, UT, United States) was fixed at one-third of the distal part of each participant’s lower leg. The evaluator asked the participants to extend their knees maximally for 3 s. All evaluations were performed by the same evaluator. The intraclass correlation coefficient (ICC) was calculated before this study. ICC (1,1) was 0.94 (95% confidence interval [CI] 0.64–0.99), and ICC (1,2) was 0.97 (95% CI 0.78–0.99). To obtain sufficient reliability, the evaluation was performed twice at each time point, and the larger value was used for comparison.

#### Gait ability

Gait ability was evaluated using the 10-m maximal walking test (MWT). The patient walked 10 m as quickly as possible. The patient was allowed to use assistive and/or orthotic devices if necessary. Two trials were performed, and the shorter time was used to compare and calculate the walking rate (WR).

#### Timed up and go test (TUG)

The TUG test was used to evaluate standing-up and sitting-down abilities. In this study, the participant was instructed to stand when the evaluator said “start”, walk at a maximal speed past the 3-m mark, turn around, walk back, and sit in the chair. The time required for the TUG test was measured using a stopwatch and recorded to the nearest second. The evaluation time point at each TUG was performed twice, and the faster time was used for statistical analysis.

#### ADL and QOL

ADL and QOL were evaluated using the Knee Injury and Osteoarthritis Outcome Score (KOOS)^11^. KOOS is a self-answered questionnaire that consists of five subscales: Pain, Other Disease-Specific Symptoms, ADL Function, Sport and Recreation Function, and Knee-Related QOL. A previous study showed that KOOS is an appropriate outcome measure in patients after TKA^12^. However, patients with severe knee OA often skipped answering the item on sports in the KOOS as it was considered irrelevant to their daily lives^13^. In this study, because the participants did not perform the motion presented in the KOOS (i.e., running and jumping) 4 weeks after TKA, this study excluded the subscale of sports in the KOOS for statistical analysis.

The pain intensity and active and passive knee extension angles were evaluated for 10 days before and after each intervention. Additionally, pain intensity, active and passive knee extension angles, IKES, MWT, and TUG were evaluated periodically before surgery and 2 and 4 weeks after TKA. The KOOS score was evaluated before and 4 weeks after TKA.

### Statistical analysis

The sample size was calculated using G-power before the study, with an alpha of 0.05, a power of 0.80, and an effect size of 0.25. The analysis of variance (ANOVA) calculation for the split-plot factorial design showed that a total of 44 patients were needed in each group, and 36 patients were needed in each group for the paired t-test.

Preoperative data for the HAL-SJ and CPT groups were compared using two-sample t-tests for age and body mass index and Pearson’s chi-square test for the ratio of women. The amount of change in pain intensity and active and passive knee extension angles before and after intervention for 10 days in each group was calculated. Mendoza’s multisample sphericity test was performed; split-plot ANOVA was performed when sphericity was assumed; and split-plot ANOVA with Greenhouse–Geisser’s epsilon correction was performed when sphericity was not assumed. The ANOVA was used to compare the number of changes over 10 days and periodic evaluations (i.e., before TKA and 2 and 4 weeks after TKA) of the HAL-SJ and CPT groups. If the interaction was significant, a two-sample t-test was used to compare the group differences at each evaluation point. A post hoc test using the Shaffer method was performed when only the main effect of the repeated-measures factor was significant. In addition, to investigate the immediate effect of the intervention, pain intensity and active and passive knee extension angles before and after intervention in each group for 10 days were compared using a paired t-test with the Bonferroni correction.

The threshold for significance was set at p = 0.05. All statistical analyses were performed using R version 3.6.3(R: R Foundation for Statistical Computing, Vienna, Austria).

## Results

Of the 76 patients, 40 were assigned to the HAL-SJ group and 36 to the CPT group. Regarding the preoperative demographic data, there were no significant differences in age, ratio of women, or body mass index between the HAL-SJ and CPT groups (Table 1).

**Table 1:** Preoperative patients demographics.

	HAL-SJ Group	CPT group	P-value
Sample number	40	36	
Age, years	74.2 (6.3)	74.0 (5.1)	0.850
Women, %	34	31	0.891
Body mass index, kg/m^2^	25.3 (2.8)	26.3 (3.3)	0.127

Age and body mass index in hybrid assistive limb single joint (HAL-SJ) and conventional physical therapy (CPT) groups are shown as mean (SD) and compared using two-sample t-tests. Additionally, the ratio of women in the two groups was compared using Pearson’s chi-square test.

### Results of the 10-day evaluation

#### Pain intensity

**Figure 3:**
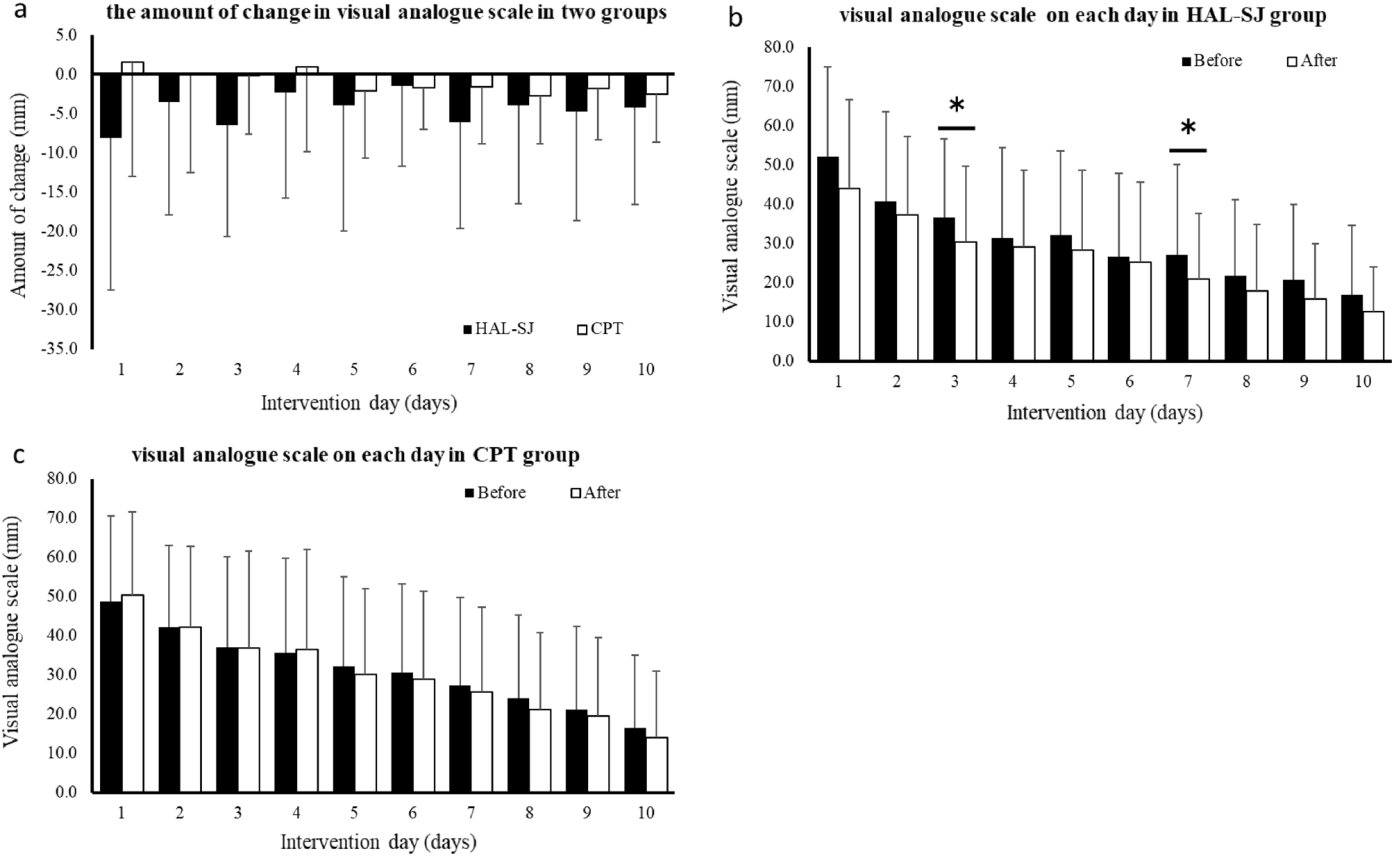
Pain intensity VAS (visual analog scale) during active knee motion for 10 days. The amount of change each day in the two groups is shown in (**a**). The before and after intervention VAS is shown in (**b**) HAL-SJ and (**c**) CPT groups. *CPT* conventional physical therapy, *HAL-SJ* single-joint hybrid assistive limb.

According to the results of the ANOVA for the change in pain intensity over 10 days (Fig. 3), the main effect of the group was significant. There were no statistically significant differences in the amount of change between groups. The HAL-SJ group showed a significant decrease immediately after the intervention on days 3 and 7. The CPT group showed no significant change after 10 days.

#### Active knee extension angle

**Figure 4:**
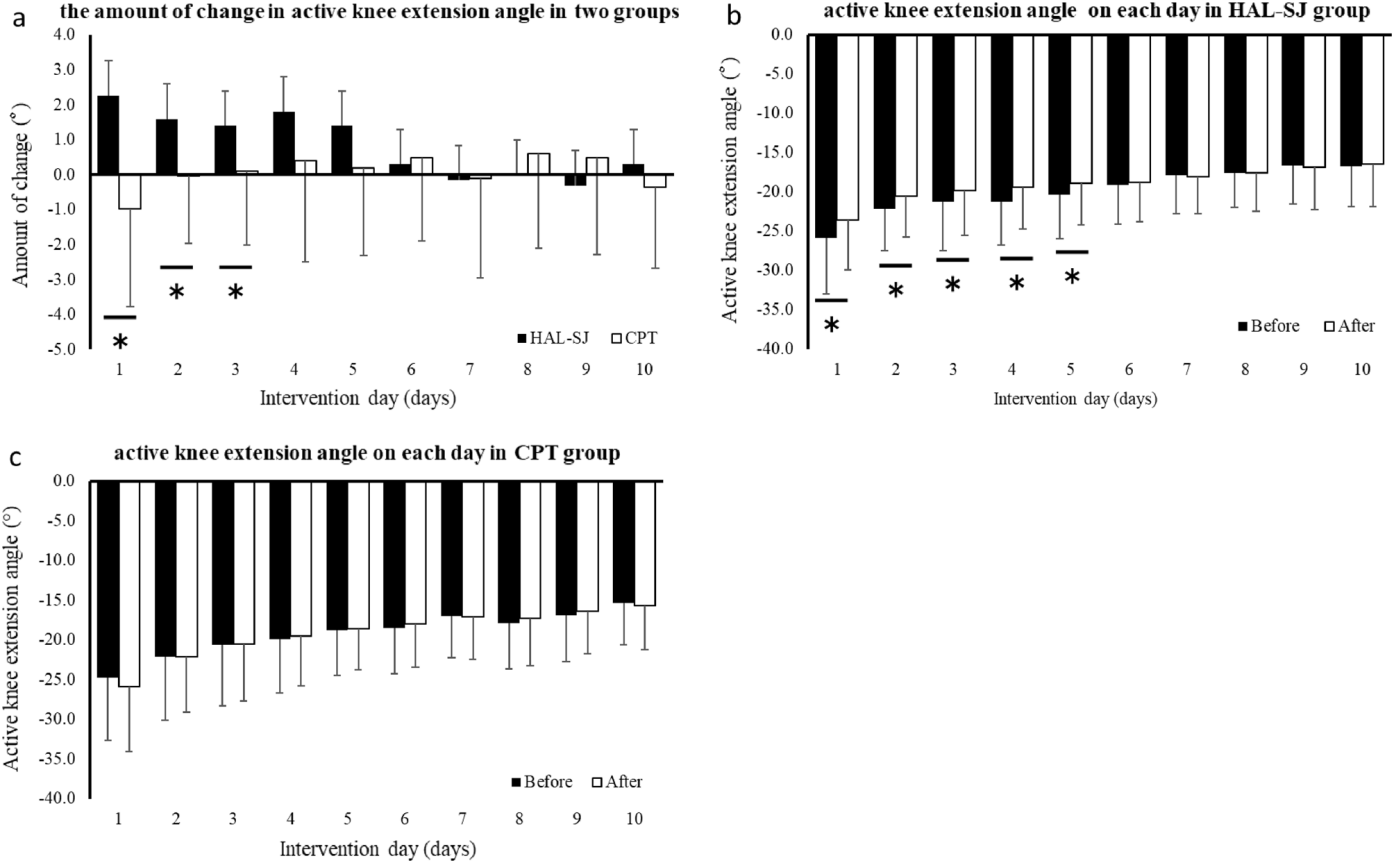
Active knee extension angle for 10 days. The amount of change each day in the two groups is shown in (**a**). The before and after intervention active knee extension angle is shown in (**b**) HAL-SJ and (**c**) CPT groups. *CPT* conventional physical therapy, *HAL-SJ* single-joint hybrid assistive limb.

Regarding the change in the active knee extension angle over 10 days, ANOVA showed a significant interaction (Fig. 4). The main effects of group and repeated measurements were also significant. When comparing the amount of change between groups, the HAL-SJ group showed significantly greater improvement than the CPT group on days 1 to 3. The HAL-SJ group significantly improved immediately after intervention on days 1 to 5. In contrast, the CPT group showed no significant change for 10 days.

#### Passive knee extension angle

**Figure 5:**
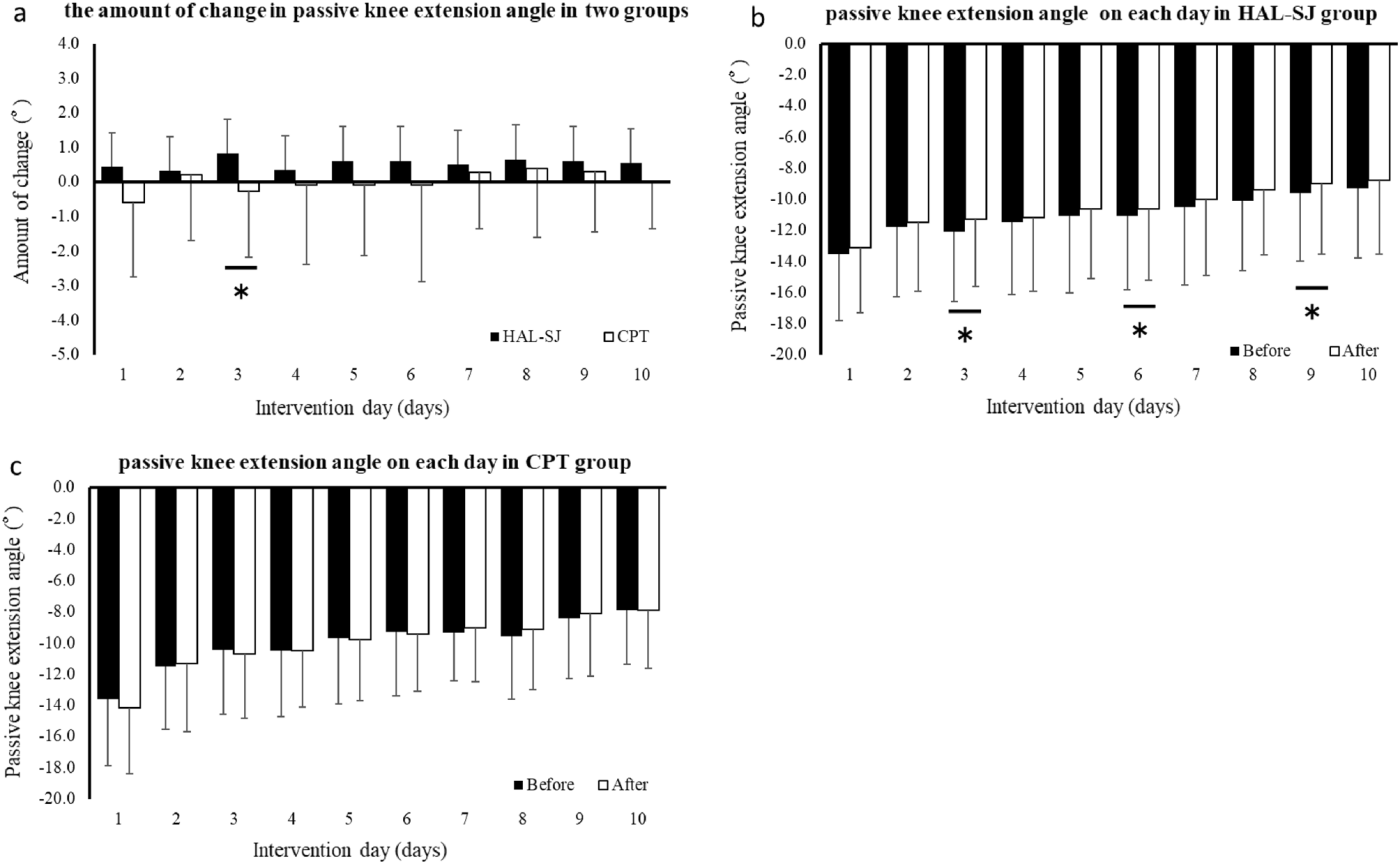
Passive knee extension angle for 10 days. The amount of change each day in the two groups is shown in (**a**). The before and after intervention passive knee extension angle is shown in (**b**) HAL-SJ and (**c**) CPT groups. *CPT* conventional physical therapy, *HAL-SJ* single-joint hybrid assistive limb.

The results of the ANOVA for the change in pain intensity over 10 days showed that the main effect of the group was significant (Fig. 5). Regarding the amount of change, the HAL-SJ group showed significantly greater improvement than the CPT group on day 3. Concerning the passive knee extension angle, the HAL-SJ group showed significant improvement immediately after the intervention on days 3, 6, and 9. By contrast, the CPT group showed no significant change after 10 days.

### All outcome measures of the periodic evaluation

For all outcome measures, only the main effect of repeated measurements was observed (Table 2). Post hoc tests were conducted only for repeated measurement effects (Table 3). Pain intensity and passive knee extension angle improved over time. The active knee extension angle, IKES, walking ability, and TUG tended to deteriorate temporarily 2 weeks after TKA and recovered to preoperative levels 4 weeks after TKA.

**Table 2:** Results of periodic evaluation of pain, knee extension angle, knee extension strength, walking function, and Knee Injury and Osteoarthritis Outcome Score (KOOS).

	Before TKA		2 weeks after TKA		4 weeks after TKA		Main effect (Group)		Main effect (Repeated Measurement)		Interaction	
	HAL-SJ	CPT	HAL-SJ	CPT	HAL-SJ	CPT	p-value	effect size (η_p_^2^)	p-value	effect size (η_p_^2^)	p-value	effect size (η_p_^2^)
Pain intensity VAS (mm)	44.2 (23.9)	48.3 (23.1)	16.5 (18.7)	22.4 (19.4)	10.6 (15.4)	10.8 (11.6)	0.333	0.013	< 0.001	0.629	0.410	0.011
Active knee extension angle (°)	− 13.5 (7.6)	− 14.2 (5.5)	− 15.7 (5.3)	− 15.0 (4.8)	− 13.5 (5.7)	− 12.7 (4.7)	0.797	0.001	0.002	0.091	0.374	0.013
Passive knee extension angle (°)	− 9.1 (7.2)	− 9.5 (5.7)	− 8.7 (4.8)	− 8.1 (3.7)	− 7.1 (4.9)	− 6.1 (3.9)	0.664	0.003	< 0.001	0.137	0.400	0.012
Isometric knee extension strength (Nm/kg)	1.0 (0.3)	1.0 (0.4)	0.7 (0.2)	0.7 (0.2)	0.9 (0.2)	0.8 (0.2)	0.616	0.003	< 0.001	0.419	0.096	0.035
Maximal walking test												
Time (s)	9.5 (3.1)	10.9 (5.2)	10.3 (2.6)	10.7 (2.8)	8.8 (2.3)	8.7 (1.9)	0.318	0.014	< 0.001	0.156	0.141	0.028
Steps (step)	21.0 (5.1)	21.6 (5.2)	22.0 (4.1)	21.5 (3.4)	20.0 (3.2)	19.6 (2.9)	0.877	< 0.001	< 0.001	0.168	0.258	0.018
Step length (m)	0.5 (0.1)	0.5 (0.1)	0.5 (0.1)	0.5 (0.1)	0.5 (0.1)	0.5 (0.1)	0.948	< 0.001	< 0.001	0.234	0.089	0.034
Walking rate (steps/s)	2.3 (0.3)	2.1 (0.4)	2.2 (0.3)	2.1 (0.3)	2.3 (0.3)	2.3 (0.3)	0.050	0.051	< 0.001	0.177	0.280	0.017
Timed up and go test (s)	10.7 (3.0)	11.4 (3.7)	11.8 (2.9)	12.3 (2.9)	10.3 (2.6)	10.1 (2.1)	0.531	0.005	< 0.001	0.256	0.225	0.020
KOOS Pain	42.1 (20.4)	40.4 (19.6)			62.4 (15.1)	68.5 (16.3)	0.522	0.006	< 0.001	0.606	0.086	0.039
Symptom	49.4 (20.9)	49.1 (20.7)			63.8 (15.6)	68.0 (16.5)	0.550	0.005	< 0.001	0.319	0.440	0.008
ADL	54.4 (19.7)	51.8 (19.9)			68.1 (17.0)	69.2 (15.5)	0.832	0.001	< 0.001	0.407	0.414	0.009
QOL	23.8 (16.4)	24.8 (15.8)			42.8 (19.8)	48.8 (22.4)	0.338	0.012	< 0.001	0.537	0.291	0.015
											Mean (SD)	

Values of the hybrid assistive limb single joint (HAL-SJ) and conventional physical therapy (CPT) groups are shown as mean (SD) and compared using split-plot design ANOVA.

*TKA* total knee arthroplasty, *VAS* visual analogue scale, *ADL* activities of daily living, *QOL* quality of life.

**Table 3:** Results of post hoc test using the Shaffer method, modified sequentially rejective Bonferroni procedure, with repeated-measures factor.

	Before TKA	2 weeks after TKA	4 weeks after TKA	p-value		
				Before vs. 2 weeks	Before vs. 4 weeks	2 weeks vs. 4 weeks
Pain intensity VAS (mm)	46.3 (23.5)	19.4 (19.2)	10.7 (13.6)	< 0.001	< 0.001	< 0.001
Active knee extension angle (°)	− 13.9 (6.7)	− 15.3 (5.1)	− 13.1 (5.2)	0.024	0.283	< 0.001
Passive knee extension angle (°)	− 9.3 (6.5)	− 8.4 (4.3)	− 6.6 (4.5)	0.176	< 0.001	< 0.001
Isometric knee extension strength (Nm/kg)	1.0 (0.4)	0.7 (0.2)	0.9 (0.2)	< 0.001	< 0.001	< 0.001
Maximal walking test						
Time (s0	10.2 (4.3)	10.5 (2.7)	8.8 (2.1)	0.500	< 0.001	< 0.001
Steps (step)	21.3 (5.1)	21.7 (3.8)	19.8 (3.1)	0.313	< 0.001	< 0.001
Step length (m)	0.5 (0.1)	0.5 (0.1)	0.5 (0.1)	0.020	< 0.001	< 0.001
Walking rate (steps/s)	2.2 (0.3)	2.1 (0.3)	2.3 (0.3)	0.066	0.002	< 0.001
Timed up and go test (s)	11.1 (3.3)	12.1 (2.9)	10.2 (2.4)	0.003	0.002	< 0.001
						Mean (SD)

*TKA* total knee arthroplasty.

## Discussion

This study investigated the effectiveness of knee extension exercises using the HAL-SJ in the acute phase after TKA. The results of this study showed an improvement in pain intensity during knee motion and knee extension angle immediately after the intervention. Even in the acute phase after TKA with severe knee pain, knee extension exercises with the HAL-SJ could contribute to immediate improvement of knee extension.

Our study showed immediate improvement in pain and knee extension angle after HAL-SJ intervention in the acute phase of TKA. We believe that the interactive biofeedback provided by HAL-SJ potentially improved muscle activity. Because muscle hypertrophy from resistance training was observed after 7–12 weeks^14^, the intervention span in this study was considered too short for muscle hypertrophy. Previous studies have shown that muscle activation failure, also known as arthrogenic muscle inhibition (AMI), occurs after TKA^3–5^. AMI occurs postoperatively with structural damage, such as TKA, and reduces output from afferent sensory receptors^15–17^. Knoop et al. reported that muscle weakness possibly impaired proprioceptive accuracy and that there was a relationship between knee pain and proprioceptive accuracy. The possibility of AMI in the acute phase after TKA may be attributed to impaired proprioceptive accuracy resulting from severe knee pain and muscle weakness.

The HAL-SJ could complement insufficient motion in the acute phase after TKA and provide patient feedback on correcting knee motion following the patient’s intent. In addition, the HAL-SJ provided patients with visual feedback on muscle activity during exercise from the controller and LED lights. A previous study has shown that exercise with HAL-SJ immediately increased cortical activation in the primary motor cortex of the ipsilesional hemisphere^18^. This study indicates that limb exercise with the HAL-SJ can affect the activity of the central nervous system. Feedback from the HAL-SJ during knee extension movements is thought to compensate for the reduced afferent sensory input from the joint and improve AMI.

This study has some limitations. First, the investigation of the mechanism of the intervention effect using HAL-SJ was insufficient. Our results indicated that the intervention effect of HAL-SJ was effective in the limited acute phase of the postoperative period. The mechanism of the intervention effect of the HAL-SJ was discussed in terms of neurological factors. However, further evaluation (i.e., proprioceptive sensation, nerve conduction velocity, and electromyography^19^) is needed to clarify this mechanism. Second, the HAL-SJ intervention only involved knee extension exercises in the sitting position. The gait pattern after TKA results in different muscle activities^20^ and poor knee joint motion^21^ compared with normal subjects, which may cause residual disability. The effectiveness of gait exercises with HAL after TKA has previously been reported^22^. As gait ability is considered more related to ADL, comparing the exercise in this study and gait exercise with HAL is necessary. Third, the use of the HAL-SJ should be explored further. In a pilot study by Tanaka et al., knee flexion exercises with HAL-SJ showed significantly greater improvement in the Oxford knee score and no significant change in ROM compared to continuous passive motion^23^. Another study demonstrated the effectiveness of knee extension exercises using the HAL-SJ after TKA^24^. However, the HAL intervention methods differed from those used in the present study (i.e., postoperative start date, frequency, and repetitions of intervention). Physical therapy in the CPT group was also not standardized, and the presence or absence of physical assistance during the intervention may affect various outcomes. In this study, neither intervention involved physical assistance by a physical therapist. Therefore, future studies should unify the HAL intervention and rehabilitation methods in the conventional group. Furthermore, although we discussed the possibility that HAL-SJ improved AMI, focal joint cooling, transcutaneous electrical nerve stimulation, and neuromuscular electrical stimulation have also been reported to be effective in treating AMI^25^. Comparative studies between these therapies and the HAL-SJ intervention for patients with AMI are needed. Finally, previous studies have shown that preoperative central sensitization (CS) is negatively correlated with improved QOL^26^ and that patients with residual CS after TKA have worse QOL, disability, and dissatisfaction^27^. CS could also have affected the results of this study, and additional studies are needed.

## Conclusion

This study investigated the effects of knee extension exercises using the HAL-SJ after TKA. Our analysis demonstrated that knee extension exercises with HAL-SJ improved knee pain and knee extension angle compared to those without HAL-SJ in the acute phase after TKA.

### Supplementary Information

Supplementary Table S1.

## Data Availability

The datasets generated and/or analyzed during the current study are available on Figshare. The URL is https://figshare.com/s/fd1b4d146d6751ccd9f6. The DOI is http://doi.org/10.6084/m9.figshare.23306126.
