# Supplementary material for: Therapeutic effect of knee extension exercise with single-joint hybrid assistive limb following total knee arthroplasty: a prospective, randomized controlled trial
Source: Sci Rep. 2024 Feb 16;14:3889. doi: 10.1038/s41598-024-53891-7 (PMC10873320; doi:10.1038/s41598-024-53891-7)
Supplement: Supplementary file 1 — Supplementary Table S1. [file 41598_2024_53891_MOESM1_ESM.docx]

**Therapeutic Effect of Knee Extension Exercise with Single-Joint Hybrid Assistive Limb Following Total Knee Arthroplasty: A Prospective, Randomized Controlled Trial**

Takaya Maeda^1^*, PT, PhD, Eiji Sasaki^2^, MD, PhD, Takayuki Kasai^3^, PT, MS, Shigesato Igarashi^1^, PT, Yuji Wakai^4^, MD, PhD. Tomoyuki Sasaki^4^, MD, PhD, Eiichi Tsuda^5^, MD, PhD, and Yasuyuki Ishibashi^2^, MD, PhD.

**Supplementary Table S1: Description of physical therapy and the interventions in each group**

|  | Intervention | Physical therapy |
| --- | --- | --- |
| Conventional physical therapy group | Active knee extension exercise without HAL-SJ.   - A physical therapist did not assist with joint movement but only provided verbal instructions. - 50 times per day. - Five sessions per week. - The total number of sessions was 10 (i.e., 10 times in 2 weeks). | The CPT and HAL-SJ groups underwent the physical therapy protocol described below. Physical therapy was performed five times per week throughout hospitalization.  POD 1: Deep vein thrombosis prophylaxis and mobilization practice.  Day 3: Gait exercise, active and passive range of motion exercise, and quadriceps muscle strength exercise*.  *Quadriceps strength exercise was started with isometric contractions in the knee extension position and transitioned to partial squatting as long as there was no pain.  Day 8: Stair-climbing exercise. |
| HAL-SL group | Active knee extension exercise with HAL-SJ.   - A physical therapist did not assist with joint movement but provided feedback on muscle activity. - HAL-SJ assisted joint motion according to voluntary muscle activity. - 50 times per day. - Five sessions per week. - The total number of sessions was 10 (i.e., 10 times in 2 weeks). |  |

HAL-SJ: hybrid assistive limb single joint, POD: postoperative day, CPT: conventional physical therapy
